# Supplementary material for: Meta-Analysis of MS-Based Proteomics Studies Indicates Interferon Regulatory Factor 4 and Nucleobindin1 as Potential Prognostic and Drug Resistance Biomarkers in Diffuse Large B Cell Lymphoma
Source: Cells. 2023 Jan 3;12(1):196. doi: 10.3390/cells12010196 (PMC9818977; doi:10.3390/cells12010196)
Supplement: Supplementary file 1 [file cells-12-00196-s001.zip › Supplementary Materials.pdf]

**Supplementary Materials**

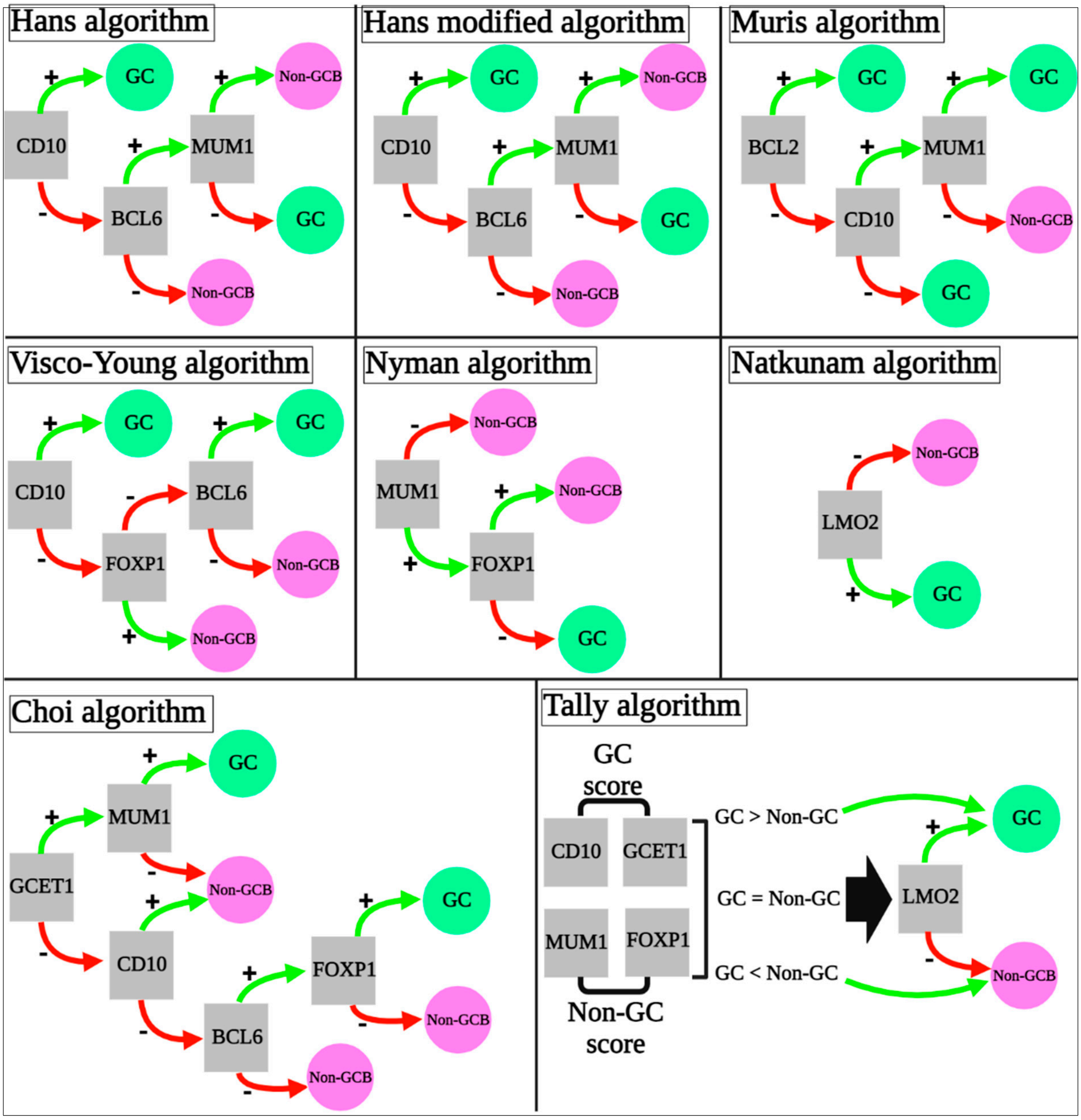


Figure S1. Common used algorithms to identify DLBCL in the clinic [1,2].

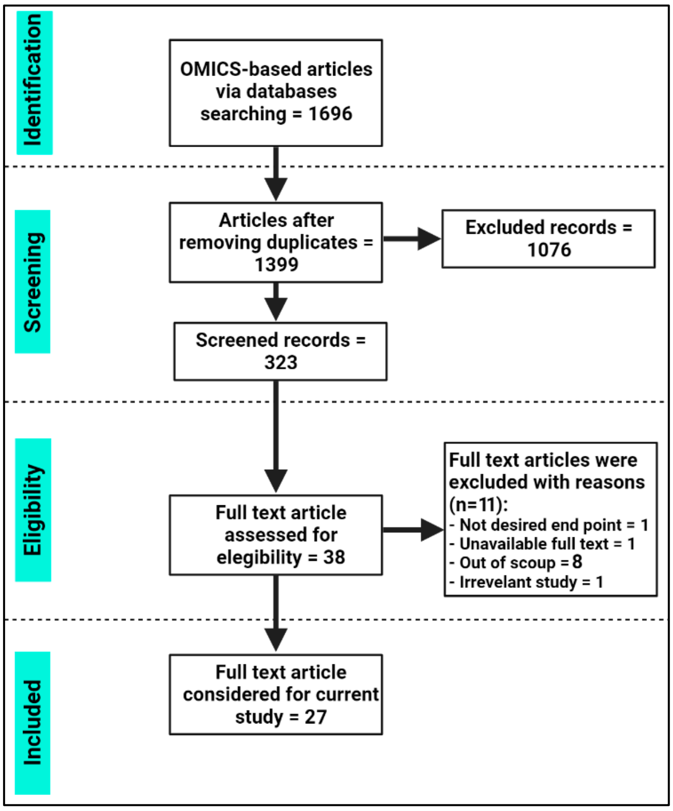


Figure S2. PRISMA flow diagram of screening studies that analysed the proteome of DLBCL with state of art mass-spectrometry.

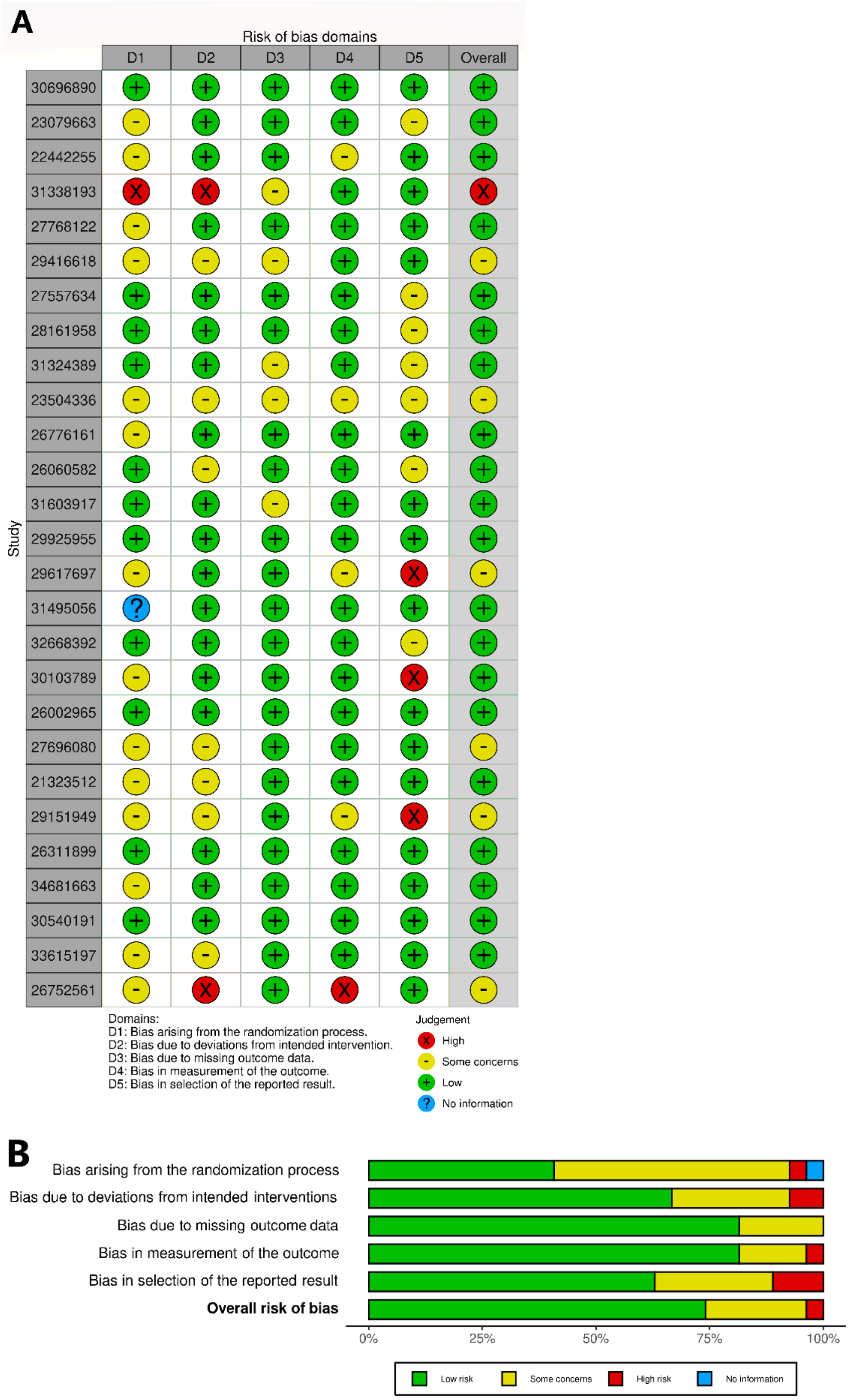


Figure S3. **(A)** Traffic light plot summaries the domain-level judgments for every single article. PUBMED IDs were used on the y-axis to show the articles; **(B)** a weighted bar plot illustrates the distribution of risk-of-bias judgments across all domains (robvis online tool was used to plot the risk of bias assessment (https://mcguinlu.shinyapps.io/robvis/).

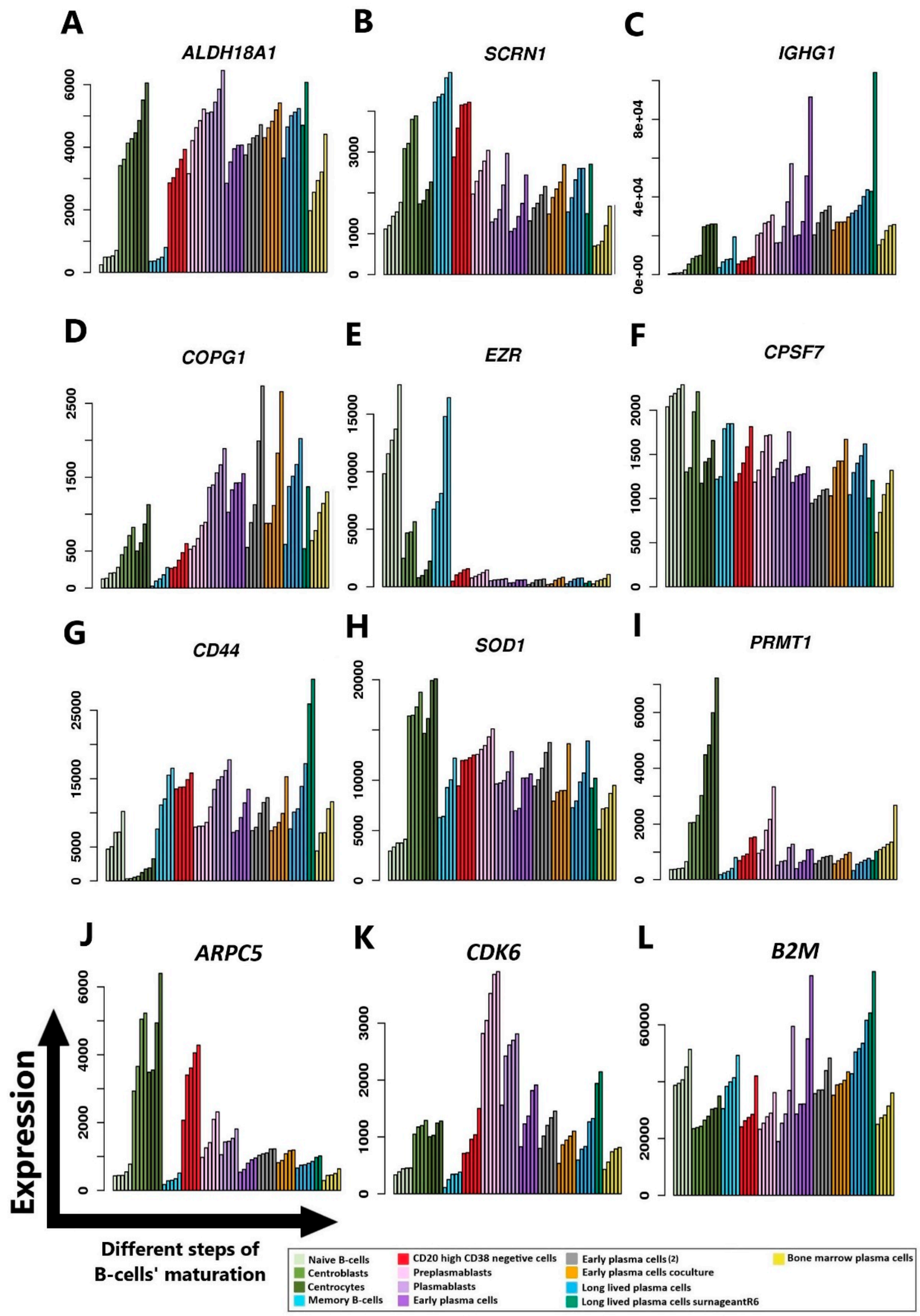


Figure S4. Expression of **12** most consistently identified proteins in healthy B cells in different stages of differentiation.

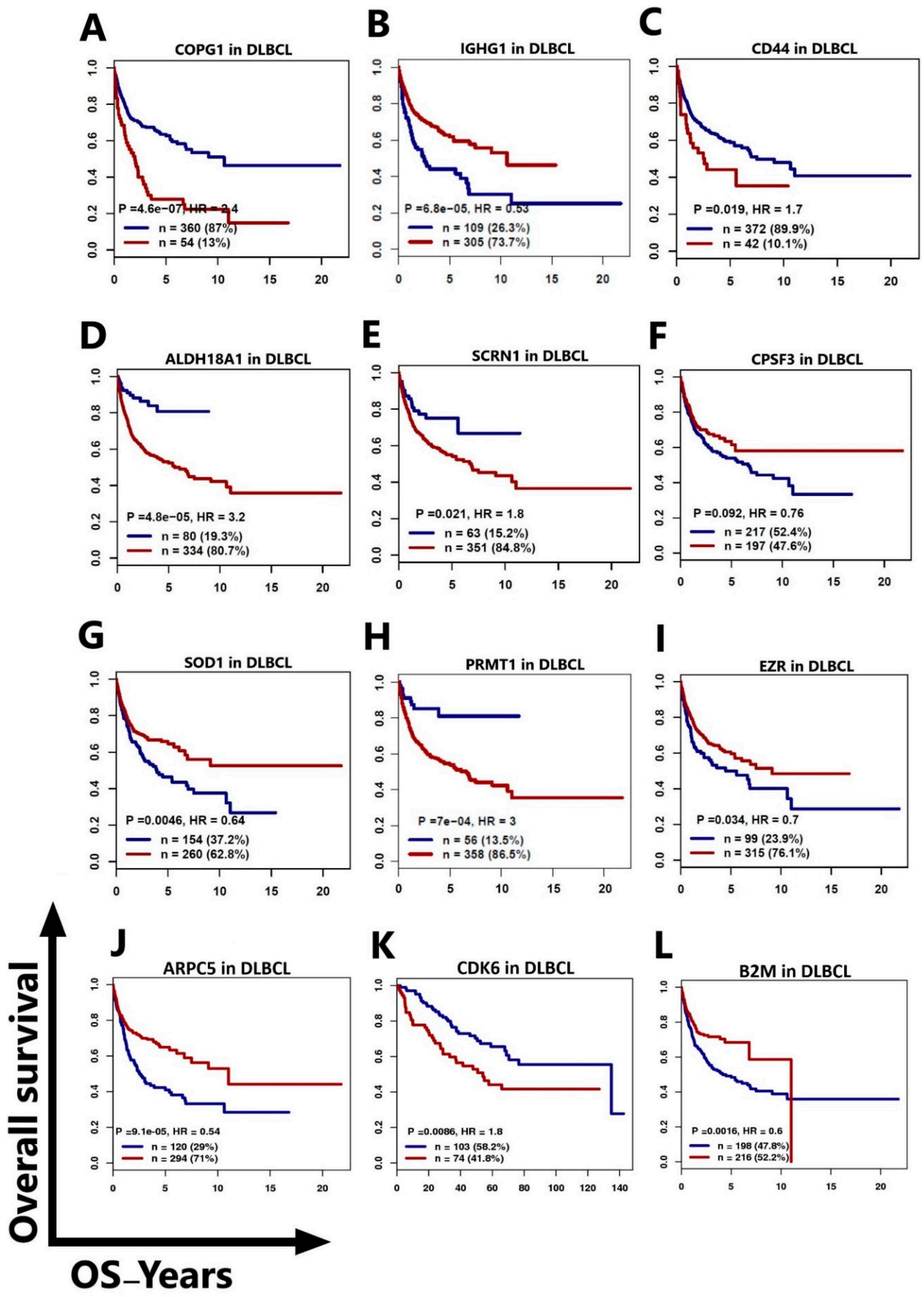


Figure S5. Impact of **12** most consistently identified proteins' expression on the overall survival of patients with DLBCL by using Genomicscape web tool (http://www.genomicscape.com/index.php). **(A-L)** the impact of ALDH18A1, IRF4, SOD1, PRMT1, CD44, CPSF7, EZR, COPG1, IGHG1, SCRN1, ARPC5, CDK6, and B2M on patients' survival. In the Genomicscape data set, patients were grouped into ABC and GCB subgroups. P-values, hazard ratios (HR), and the level of expression signal are shown (blue indicate low expression and red high expression).

# Epstein barr virus infection

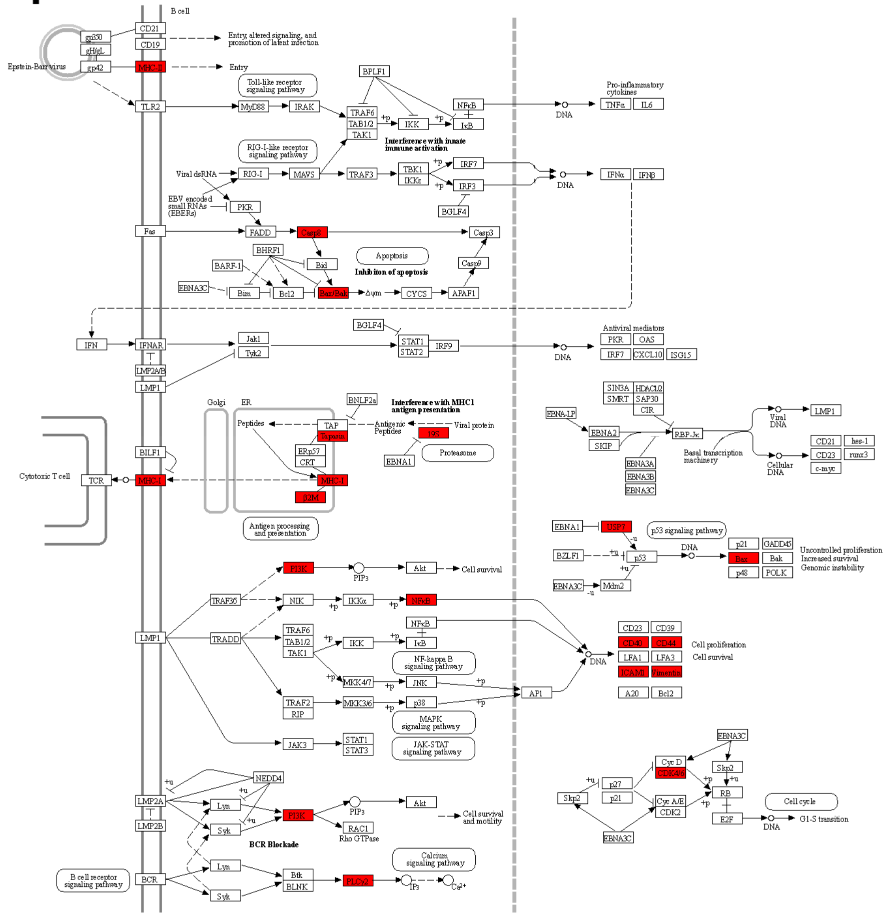

Figure S6. Epstein-bar virus (EBV) infection pathway based on recognized proteins from the group of proteins repeated in 4≤ articles. The identified proteins are represented in red. Pathview R package was used for plotting and the red colour is just used to show the identified proteins in the pathway.

Table S1. Common used algorithms to identify DLBCL in the clinic [3,4].

| Ann Arbor Stage | Involvement |
|---|---|
| 1 | A single site of extra-lymphatic involvement without the involvement of lymph nodes |
| 2 | Extra-lymphatic involvement, regional lymph node involvement, and possible additional lymph nodes on the same side of the diaphragm |
| 3 | Extra-lymphatic involvement, regional lymph node involvement, lymph nodes on both sides of the diaphragm, no involvement of spleen |
| 4 | Involvement of one or more extra-lymphatic sites or metastasis to liver, bone marrow, lungs, or cerebrospinal fluid |

Table S2. Lugano classification [5].

| Lugano stage | Involvement |
|---|---|
| **Stage I** | Tumour confined to GI tract<br>Single primary site or multiple, non-contiguous lesions |
| **Stage II** | Tumour extending into the abdomen from primary GI site<br>Nodal involvement |
| **Stage II1** | local (perigastric in cases of gastric lymphoma and para-intestinal for intestinal lymphoma) |
| **Stage II2** | distant (mesenteric in the case of an intestinal primary, otherwise; para-aortic para-caval, pelvic, inguinal) |
| **Stage IIE** | Penetration of serosa to involve adjacent organs or tissues (enumerate actual site of involvement, e.g.IIE [pancreas] IIE [large intestine], IIE[post abdominal wall]) |
| **Stage IV** | Disseminated extranodal involvement, or, a GI tract lesion with supra-diaphragmatic nodal involvement |

Table S3. Modified Ann Arbor Staging of NHL [6].

| Modified Ann Arbor Stage | Involvement |
|---|---|
| **I** | Involvement of a single lymph node region |
| **II** | Involvement of ≥2 lymph node regions on the same side of the diaphragm |
| **III** | Involvement of lymph node regions on both sides of the diaphragm |
| **IV** | Multifocal involvement of ≥1 extra lymphatic site ± associated lymph nodes or isolated extra lymphatic organ involvement with distal nodal involvement |

Table S4. Paris TNM Staging Systems for lymphoma [7].

| Paris TNMB stage | Involvement |
|---|---|
| **Tx** | Tx Lymphoma extent not specified |
| **TO** | TO No evidence of lymphoma |
| **T1** | T1 Lymphoma confined to the mucosa/submucosa |
| **T1m** | T1m Lymphoma confined to the mucosa |
| **T1sm** | T1sm Lymphoma confined to the submucosa |
| **T2** | T2 Lymphoma infiltrates muscularis propria or subserosa |
| **T3** | T3 Lymphoma penetrates serosa (visceral peritoneum) without invasion of adjacent structures |
| **T4** | T4 Lymphoma invades adjacent structures or organs |
| **Nx** | Nx Involvement of lymph nodes not assessed |
| **N0** | N0 No evidence of lymph node involvement |
| **N1** | N1 Involvement of regional lymph nodes |
| **N2** | N2 Involvement of intra-abdominal lymph nodes beyond the regional area |
| **N3** | N3 Spread to extra-abdominal lymph nodes |
| **Mx** | Mx Dissemination of lymphoma not assessed |
| **M0** | M0 No evidence of extranodal dissemination |
| **M1** | M1 Non-continuous involvement of separate sites in the gastrointestinal tract (e.g., stomach and rectum) |
| **M2** | M2 Non-continuous involvement of other tissues (e.g., peritoneum, pleura) or organs (e.g., tonsils, parotid gland, ocular adnexa, lung, liver, spleen, kidney, breast, etc.) |
| **Bx** | Bx Involvement of bone marrow not assessed |
| **B0** | B0 No evidence of bone marrow involvement |
| **B1** | B1 Lymphomatous infiltration of bone marrow |
| **TNM** | TNM Clinical staging: status of tumor, node, metastasis, bone marrow |
| **pTNMB** | pTNMB Histopathological staging: status of tumor, node, metastasis, bone marrow |
| **pN** | The histological examination will ordinarily include 6 or more lymph nodes |

References:


1. Hwang, H.S.; Yoon, D.H.; Suh, C.; Park, C.-S.; Huh, J. Prognostic value of immunohistochemical algorithms in gastrointestinal diffuse large B-cell lymphoma. Blood Res. 2013, 48, 266–273, https://doi.org/10.5045/br.2013.48.4.266.
2. Meyer, P.N.; Fu, K.; Greiner, T.C.; Smith, L.M.; Delabie, J.; Gascoyne, R.D.; Ott, G.; Rosenwald, A.; Braziel, R.M.; Campo, E.; et al. Immunohistochemical Methods for Predicting Cell of Origin and Survival in Patients With Diffuse Large B-Cell Lymphoma Treated With Rituximab. J. Clin. Oncol. 2011, 29, 200–207, https://doi.org/10.1200/jco.2010.30.0368.
3. Moormeier, J.A.; Williams, S.F.; Golomb, H.M. The staging of non-Hodgkin's lymphomas. Semin. Oncol. 1990, 17, 43–50.
4. Zapater, E.; Bagán, J.V.; Carbonell, F.; Basterra, J. Malignant lymphoma of the head and neck. Oral Dis. 2010, 16, 119–128, https://doi.org/10.1111/j.1601-0825.2009.01586.x.
5. Rohatiner, A.; D'Amore, F.; Coiffier, B.; Crowther, D.; Gospodarowicz, M.; Isaacson, P.; Lister, T.; Norton, A.; Salem, P.; Shipp, M.; et al. Report on a workshop convened to discuss the pathological and staging classifications of gastrointestinal tract lymphoma. Ann. Oncol. 1994, 5, 397–400, https://doi.org/10.1093/oxfordjournals.annonc.a058869.
6. National Cancer Institute sponsored study of classifications of non-Hodgkin's lymphomas: summary and description of a working formulation for clinical usage. Cancer 1982., 49, 2112–35.
7. Ruskone-Fourmestraux, A.; Dragosics, B.; Morgner, A.; Wotherspoon, A.; De Jong, D. Paris staging system for primary gastrointestinal lymphomas. Gut 2003, 52, 912–913, https://doi.org/10.1136/gut.52.6.912.
